# The BEYONDD Project: Preliminary Findings within a Community‐Based Sample

**DOI:** 10.1002/alz70856_107586

**Published:** 2026-01-09

**Authors:** Eden V. Barragan, Hilary W. Heuer, Rachel L. Nosheny, Monica R. Camacho, Krista Navarra, Paul S. Aisen, Annalise Rahman‐Filipiak, J. Scott Roberts, Adeyinka Ajayi, Omobolanle Ayo, Se Jun Cho, Julia E. Culhane, Kaori Kubo Germano, Vanessa A. Guzman, Michelle Higuera, Melissa Sotelo, Desiree Byrd, Monica G Rivera Mindt, Gil D. Rabinovici, Adam L. Boxer

**Affiliations:** ^1^ Memory and Aging Center, Weill Institute for Neurosciences, University of California, San Francisco, San Francisco, CA, USA; ^2^ University of California, San Francisco, San Francisco, CA, USA; ^3^ VA Advanced Imaging Research Center, San Francisco Veterans Affairs Medical Center, San Francisco, CA, USA; ^4^ Northern California Institute for Research and Education (NCIRE), San Francisco, CA, USA; ^5^ Alzheimer's Therapeutic Research Institute, University of Southern California, San Diego, CA, USA; ^6^ Michigan Alzheimer's Disease Research Center, Ann Arbor, MI, USA; ^7^ University of Michigan School of Public Health, Ann Arbor, MI, USA; ^8^ Icahn School of Medicine at Mount Sinai, New York, NY, USA; ^9^ Fordham University, New York, NY, USA; ^10^ Memory and Aging Center, UCSF Weill Institute for Neurosciences, University of California, San Francisco, San Francisco, CA, USA; ^11^ CUNY, Queens College, Queens, NY, USA; ^12^ Memory and Aging Center, Weill Institute for Neurosciences, University of California San Francisco, San Francisco, CA, USA

## Abstract

**Background:**

Early onset dementia (EOD) affects people at the peak of personal and professional responsibilities and economic productivity. Alzheimer's disease (AD) and Frontotemporal Dementia (FTD) are the most common EOD etiologies, but have not been studied in a sample representative of the general US population. Multiple barriers impede research participation for many groups, but community‐engaged research (CER) strategies to enhance recruitment may make research participation more accessible and convenient for all.

**Method:**

BEYONDD is an NIH‐funded, community‐based study focused on understanding the etiology of EOD, uses CER strategies such as remote assessments and return of research results as tools for enhancing sample representativeness. Participants are recruited using social media and local in‐person CER strategies and screened via an online platform for eligibility (age 40‐64, with concerns about cognitive or behavioral function). Remote completion of online questionnaires, cognitive testing, and an in‐home blood draw for standard labs, Aβ42/40 and *p*‐tau217 ratios, and plasma NfL comprise the initial visit. Participants are invited for more comprehensive onsite evaluation at one of 7 BEYOND in‐clinic sites, followed by tailored referral to other NIH‐funded research programs. Participants can learn their results remotely or in person.

**Result:**

Using a novel, CER‐based approach for social media ad deployment, BEYONDD has recruited over 1700 potential participants across the US and Puerto Rico; over half (*n* = 881) completed the online screening survey. Of the 206 participants enrolled in the online procedures, 80% (*n* = 165) were women and most were Latino (*n* = 83; 40%) or Black (*n* = 68; 33%) and over a quarter (*n* = 74; 36%) reported less than 16 years of education. We have completed over 95 blood draws across 17 states and invited participants for onsite visits. Results have been shared with 22 participants (10 on‐site, 12 remotely). Preliminary analyses reveal a high Aβ42/40 positivity rate (∼30%) and the most prevalent routine lab abnormalities include: LDL‐Cholesterol (63%), homocysteine (36%), hs‐CRP (35%) and HgbA1C (33%). Abnormal *p*‐tau217 ratios (*n* = 4; 5%) and APS2 scores (*n* = 3; 4%) have also been identified in this community‐based sample.

**Conclusion:**

Preliminary results suggest feasibility and acceptability of this innovative CER approach in a more representative sample of the US population.

